# Mitochondrial transfer - a novel promising approach for the treatment of metabolic diseases

**DOI:** 10.3389/fendo.2023.1346441

**Published:** 2024-01-19

**Authors:** Ruijing Chen, Jun Chen

**Affiliations:** ^1^ Department of Endocrinology, Qilu Hospital, Shandong University, Jinan, Shandong, China; ^2^ Institute of Endocrine and Metabolic Diseases of Shandong University, Jinan, Shandong, China; ^3^ Key Laboratory of Endocrine and Metabolic Diseases, Shandong Province Medicine and Health, Jinan, Shandong, China; ^4^ Jinan Clinical Research Center for Endocrine and Metabolic Diseases, Jinan, Shandong, China

**Keywords:** mitochondria, mitochondrial transfer, transplantation, metabolic diseases, therapy

## Abstract

Metabolic disorders remain a major global health concern in the 21st century, with increasing incidence and prevalence. Mitochondria play a critical role in cellular energy production, calcium homeostasis, signal transduction, and apoptosis. Under physiological conditions, mitochondrial transfer plays a crucial role in tissue homeostasis and development. Mitochondrial dysfunction has been implicated in the pathogenesis of metabolic disorders. Numerous studies have demonstrated that mitochondria can be transferred from stem cells to pathologically injured cells, leading to mitochondrial functional restoration. Compared to cell therapy, mitochondrial transplantation has lower immunogenicity, making exogenous transplantation of healthy mitochondria a promising therapeutic approach for treating diseases, particularly metabolic disorders. This review summarizes the association between metabolic disorders and mitochondria, the mechanisms of mitochondrial transfer, and the therapeutic potential of mitochondrial transfer for metabolic disorders. We hope this review provides novel insights into targeted mitochondrial therapy for metabolic disorders.

## 1 Introduction

Metabolic disorders, including diabetes, non-alcoholic fatty liver disease (NAFLD), obesity, hyperlipidemia, and gout, are major health concerns (1, 2). Diabetes has become one of the most prevalent health disparities, with an estimated 537 million adults aged 20-79 living with diabetes worldwide in 2021. This accounts for 10.5% of the global population in that age group. It is projected to rise to 643 million (11.3%) by 2030 and 783 million (12.2%) by 2045 (3). NAFLD, a common metabolic disorder, encompasses a spectrum of liver abnormalities from non-alcoholic fatty liver (NAFL) to non-alcoholic steatohepatitis (NASH), which can progress to cirrhosis and liver cancer (4–6).

Mitochondria are semi-autonomous, semi-self-replicating, highly dynamic organelles with their own circular double-stranded DNA molecules (7–9). They are responsible for coordinating cellular energy production and are involved in calcium signaling, cell growth and differentiation, cell cycle control, and cell death processes (8, 10–12). Mitochondrial dysfunction is a common underlying pathophysiological mechanism in many diseases, characterized by the generation of reactive oxygen species and accumulation of mitochondrial DNA (mtDNA) damage, leading to mitochondrial dysfunction (13–15).

In 2004, Rustom et al. discovered the existence of mitochondrial transport through tunneling nanotubes (TNTs) (16). Subsequent studies demonstrated the widespread ability of mammalian cells to acquire organelles from other cells, including the transfer of mitochondria between mammalian cells (17–21). Further research by Rustom et al. found that mesenchymal stem cells transfer mitochondria to recipient cells, rescuing injured cells, improving oxidative phosphorylation, increasing ATP production, and restoring mitochondrial function (22). Therefore, mitochondrial transfer holds potential therapeutic effects for metabolic disorders and their complications (23–25). This article provides a comprehensive review of research progress on mitochondrial transfer in the treatment of metabolic disorders, offering valuable insights into this field.

## 2 Association between metabolic disorders and mitochondria

Abnormal mitochondrial function contributes to pathological changes in cellular energy metabolism, disruption of fatty acid metabolism, and increased oxidative stress, leading to the development of metabolic disorders such as type 2 diabetes, obesity, dyslipidemia, and cardiovascular diseases. This section reviews the association between diabetes, obesity, NAFLD, and mitochondrial dysfunction.

### 2.1 Diabetes

Type 2 diabetes mellitus (T2DM) is associated with alterations in oxidative metabolism in insulin-responsive tissues. T2DM is characterized by reduced mitochondrial oxidative phosphorylation capacity and decreased mitochondrial content in skeletal muscle cells and hepatocytes. Insulin resistance occurs many years before the onset of T2DM. Acquired insulin resistance is associated with decreased mitochondrial activity in response to insulin stimulation, while inherited insulin resistance is typically linked to reduced basal mitochondrial activity, possibly due to decreased mitochondrial content (26). Peroxisome proliferator-activated receptor γ coactivator-1α (PGC-1α), a member of the transcriptional coactivator family, plays a central role in the regulation of cellular energy metabolism (27). Activation of PGC-1α has been shown to increase mitochondrial capacity for oxidative phosphorylation, restore mitochondrial superoxide production, promote insulin secretion in pancreatic β-cells, enhance insulin sensitivity in skeletal muscle and liver, and prevent diabetes microvascular complications (28). In T2DM, decreased expression of PGC-1α and its target genes leads to decreased mitochondrial ATP production (29) and increased ROS generation (30). Similar studies have also suggested that in some tissues associated with diabetic complications, exposure to excessive glucose or nutrient stress leads to decreased mitochondrial superoxide production, oxidative phosphorylation, and mitochondrial ATP generation (28). Obesity, T2DM, and aging are associated with impaired skeletal muscle oxidative capacity, reduced mitochondrial content, and decreased oxidative phosphorylation rates (31). Mitochondrial dysfunction may accelerate the progression of insulin resistance and subsequent organ dysfunction by increasing reactive oxygen species production, while lifestyle and pharmacological interventions can improve insulin resistance by enhancing oxidative phosphorylation capacity and mitochondrial content in early-stage diabetes cases (26).

### 2.2 NAFLD

NAFLD is considered a mitochondrial disease (32). In mouse models of simple steatosis or NASH, mitochondrial ATP synthesis is reduced, and mitochondria cannot oxidize sufficient fatty acids, leading to hepatic fat accumulation. Additionally, high-fat diet feeding leads to excessive calcium uptake by liver mitochondria, disrupting respiration and increasing ROS production (33). Mitochondrial fragmentation, mediated by Drp1 and MFF, has been shown to increase fatty acid oxidation in adipocytes and serves as a compensatory mechanism against nutrient overload (34, 35). Studies have demonstrated that hepatocyte-specific loss of mitochondrial fusion protein 2 (Mfn2) exacerbates NAFLD progression, inflammation, and hyperglycemia in high-fat diet-fed mice (36). Mitochondrial fragmentation may drive steatosis and NASH by reducing fatty acid oxidation. Moreover, mitochondrial fragmentation can activate mitophagy for the clearance of damaged mitochondria (34). It has been demonstrated that increased levels of fatty acids and ceramides raise the lysosomal pH in hepatocytes, impairing lysosomal function and halting autophagy, leading to the accumulation of damaged mitochondria (37). A recent study showed that hepatocyte-specific loss of Parkin, which selectively targets damaged mitochondria for mitophagy, exacerbates hepatic steatosis and insulin resistance in high-fat diet-fed mice (38). Therefore, reduced mitochondrial engulfment in NASH liver may be due to the failure or dormancy of mechanisms for the removal of damaged mitochondria, allowing the presence of a large number of damaged mitochondria (39).

### 2.3 Obesity

Mitochondria are closely associated with fatty acid synthesis metabolism and adipocyte function, thus influencing the development of obesity. Similar to diabetes, mitochondrial biogenesis is also reduced in obese individuals (40). Research by Forner et al. revealed that mitochondria in white and brown fat utilize specific isoforms for fatty acid degradation and biosynthesis. Mitochondrial isoforms Gpam, AGPAT2, AGPAT3, DGAT1, Agk, and Acp6 are enriched in the white mitochondrial fraction, and their activities are closely related to triglyceride biosynthesis in adipocytes (41). Studies have shown that decreased mitochondrial transfer from adipocytes to macrophages is associated with obesity in mice (42). Another hallmark of metabolically unhealthy adipocytes is mitochondrial dysfunction (43), which affects adipogenesis, adipokine secretion, lipid turnover, and lipolysis (44). Evidence suggests that obesity is associated with mitochondrial dysfunction in white adipose tissue, characterized by decreased mitochondrial DNA (mtDNA) content, decreased expression of electron transport chain (ETC) genes, impaired mitochondrial oxidative capacity, and elevated reactive oxygen species (ROS) levels (45). Additionally, Yu et al. reported that dysregulation of amyloid precursor protein impairs mitochondrial function in adipose tissue and promotes obesity (46).

## 3 Mechanisms of intercellular mitochondrial transfer

Since the discovery of mitochondrial transfer from endothelial progenitor cells to cardiomyocytes by Masamichi Koyanagi in 2005 (47), numerous scientists have identified additional evidences of intercellular mitochondrial transfer and investigated their underlying molecular mechanisms. Most studies indicate that mitochondria can be transferred through TNTs, extracellular vesicles (EVs), gap junction channels (GJCs), cell fusion, and mitochondrial extrusion. This section provides a review of the identified modes of mitochondrial transfer to date.

### 3.1 Mitochondrial transfer via tunneling nanotubes

**Figure 1:**
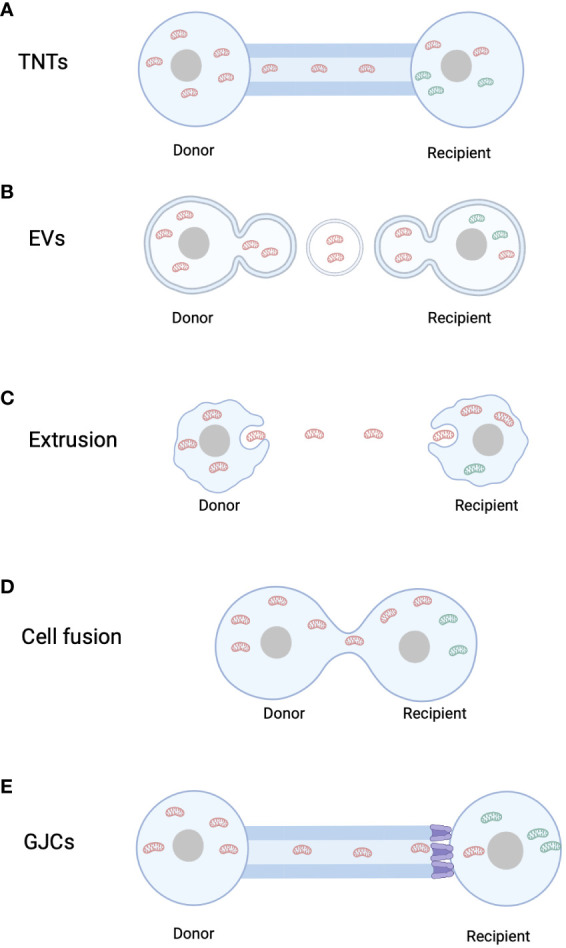
Mitochondrial transfer pathways. **(A)** Tunnel Nanotubes (TNTs). **(B)** Extracellular Vesicles (EVs). **(C)** Mitochondrial Extrusion. **(D)** Cell fusion. **(E)** Gap junction channels. Red mitochondria refer to mitochondria originating from the donor cell, whereas green mitochondria denote mitochondria originating from the recipient cell.

In 2004, Rustom et al. observed the presence of TNTs in rat pheochromocytoma PC12 cells using 3D microscopy (16). TNTs are intercellular structures with diameters ranging from 50 to 200 nm. Subsequently, the formation of TNTs between endothelial progenitor cells and cardiomyocytes was demonstrated using MitoTracker labeling (47). Since the discovery of mitochondrial transfer through TNTs in mammalian cells, increasing evidence has emerged regarding their roles in apoptosis and tumorigenesis (**Figure 1A**). Wang and Gerdes found that mitochondrial transfer via TNTs could rescue apoptosis in UV-treated PC12 cells (48). Co-culturing UV-treated PC12 cells with untreated PC12 cells resulted in the formation of a novel type of TNT, through which mitochondria from healthy cells were transferred to UV-treated PC cells, protecting them from apoptosis (48). In 2022, Yang et al. demonstrated that marrow stromal cells could acquire mitochondria from rotenone-induced mitochondrial dysfunction myeloid cells through TNTs in co-culture systems, preventing mitochondrial dysfunction and apoptosis in recipient myeloid cells (49). Recent research has also revealed that tumor cells can “steal” mitochondria from immune cells through TNTs, highlighting the clinical relevance of this phenomenon (50). As mitochondria contain a separate mtDNA, their transfer through TNTs not only directly influences the metabolism of recipient cells but also affects the mitochondrial genome of the recipient cells. Valdebenito et al. found that TNTs were formed between GBM cells and primary astrocytes under co-culture and stress conditions. Mitochondria from tumor cells were transferred to primary astrocytes, and the transferred mtDNA contained genetic variants that altered the metabolism of the recipient cells (51).

### 3.2 Mitochondrial transfer via extracellular vesicles

Cells can communicate with adjacent or distant cells through the secretion of EVs. EVs can be categorized into two types: microvesicles, which bud from the plasma membrane and have diameters ranging from 100 nm to 1000 nm, and exosomes, which are smaller vesicles (diameter < 150 nm) enriched with nuclear-derived components. While exosomes cannot carry intact mitochondria due to their small size, larger EVs have the capacity to transport complete mitochondria. Once attached to target cells, EVs can induce signal transduction through receptor-ligand interactions and can be internalized through endocytosis and/or phagocytosis or fuse with the target cell membrane, delivering their cargo into the cytoplasm (52). In 2012, Islam et al. demonstrated that bone marrow-derived mesenchymal stem cell (BMSC)-derived EVs can deliver mitochondria to damaged alveolar epithelial cells in a mouse model of lipopolysaccharide-induced acute lung injury, thereby preventing acute lung injury (53). Since then, more evidence of mitochondrial transfer via vesicles has been discovered (**Figure 1B**). Phinney et al. found that bone marrow-derived mesenchymal stem cells can transfer mitochondria to macrophages through microvesicles (54). Additionally, Peruzzotti-Jametti et al. demonstrated that EVs secreted by neural stem cells can rescue mitochondrial function in L929 Rho0 cells with mtDNA defects and integrate into the mitochondrial network of host inflammatory macrophages (55). Not only stem cells, but various cell types have been shown to transfer mitochondria through EVs to exert various biological functions. Hayakawa et al. found that astrocytes release extracellular mitochondrial particles after a stroke, which enter post-stroke neurons and promote ATP production and neuronal recovery (56). Hough et al. discovered that extracellular vesicles mediate mitochondrial transfer from airway myeloid progenitors to T cells (57). Collectively, these studies suggest that mitochondrial transfer through various types of extracellular vesicles is an effective mechanism for restoring cellular functionality.

### 3.3 Mitochondrial transfer via mitochondrial extrusion or internalization

In 2008, Nakajima et al. first described mitochondrial extrusion. Mitochondrial extrusion is a unique form of cell death mediated by tumor necrosis factor α, as it is not observed in cell death induced by the genotoxic drug cisplatin. During mitochondrial extrusion, fragmented mitochondria are detached from the cell via cell membrane vesicles, which engulf the mitochondrial fragments. When the membrane enclosing the mitochondria fuses with the cell membrane, the exposed mitochondria are released into the extracellular space, inducing cell death (**Figure 1C**). Mitochondrial extrusion and membrane blebbing require intact cellular scaffolds composed of actin and microtubules. Under physiological conditions, red blood cells extrude mitochondria during maturation, while under pathological conditions, cytoplasmic vesicles engulf mitochondrial fragments injected into mice with anti-Fas antibodies are extruded from hepatocytes (58). Additionally, Lyamzaev et al. found that mitochondria cluster near the cell nucleus, forming mitochondrial clusters that separate from the cytoplasm through membrane fission, termed “mitoptotic bodies,” which are then released from the cell (59).

Naked mitochondria or mitochondrial components can also be extruded and internalized without a carrier, a process known as blebbing and internalization (60). Kitani et al. demonstrated that isolated mitochondria can be internalized into cells through simple co-incubation using genetically labeled mitochondria. Mitochondrial internalization significantly improved mitochondrial function in cells depleted of mtDNA, and this effect was sustained for several days (61).

### 3.4 Mitochondrial transfer via cell fusion

While cell fusion can mediate mitochondrial transfer, related research in this area is limited. Cell fusion refers to the process whereby two independent cells merge their cell membranes, share organelles and cytoplasm, while maintaining intact cell nuclei. Permanent cell fusion results in the sharing of cytoplasm and the formation of a unique nuclear configuration, whereas partial cell fusion allows transient cell communication or exchange of organelles, such as mitochondria (23). Adrien et al. demonstrated that human mesenchymal stem cells can reprogram myocytes into an immature state through partial cell fusion and mitochondrial transfer (**Figure 1D**) (62). Co-culture of cardiac myocytes with human multipotent adipose-derived stem cells allowed partial cell fusion, facilitating the exchange of materials and mitochondria (62).

### 3.5 Mitochondrial transfer via gap junction channels

Gap junction channels (GJCs) are intercellular channels formed by the docking of two adjacent hemichannels (HCs) located in the plasma membranes of neighboring cells. Each HC is composed of six connexin (Cx) subunits, forming a hollow tubular structure (63). Direct transfer of mitochondria through GJCs is not possible due to the much smaller pore size of GJCs compared to mitochondria (63, 64). However, Alarcon-Martinez et al. discovered interstitial primitive TNTs (IP-TNTs), which consist of both TNTs and a distal Cx43-GJC connecting two recipient cells. Live imaging revealed the presence of mitochondria within IP-TNTs and their movement within these structures (**Figure 1E**) (65). However, mitochondrial transfer did not occur between recipient cells, indicating that GJCs cannot directly mediate mitochondrial transfer (65). Nonetheless, evidence suggests that GJCs based on Cx43 may play a role in TNT-mediated mitochondrial transfer. Norris used 3D electron microscopy and immunogold labeling of Cx43 to reveal that recipient cell mitochondria can internalize through Cx43-based GJC-mediated endocytosis into double-membrane autophagosome-like GJCs (66). Yao et al. observed that Cx43 in TNTs between induced pluripotent stem cell-derived mesenchymal stem cells (iPSC-MSCs) and BEAS2B cells was crucial for TNT formation, and knocking down Cx43 expression significantly affected TNT formation and reduced mitochondrial transfer between the two cell types (67). In contrast, enhanced Cx43 expression promoted mitochondrial transfer from astrocytes to neurons through TNT-like structures (68). These findings suggest that Cx43-based GJCs may contribute to TNT-mediated mitochondrial transfer.

In summary, while GJCs cannot directly mediate mitochondrial transfer, they may facilitate TNT-mediated mitochondrial transfer.

## 4 Methods of mitochondrial transplantation

**Figure 2:**
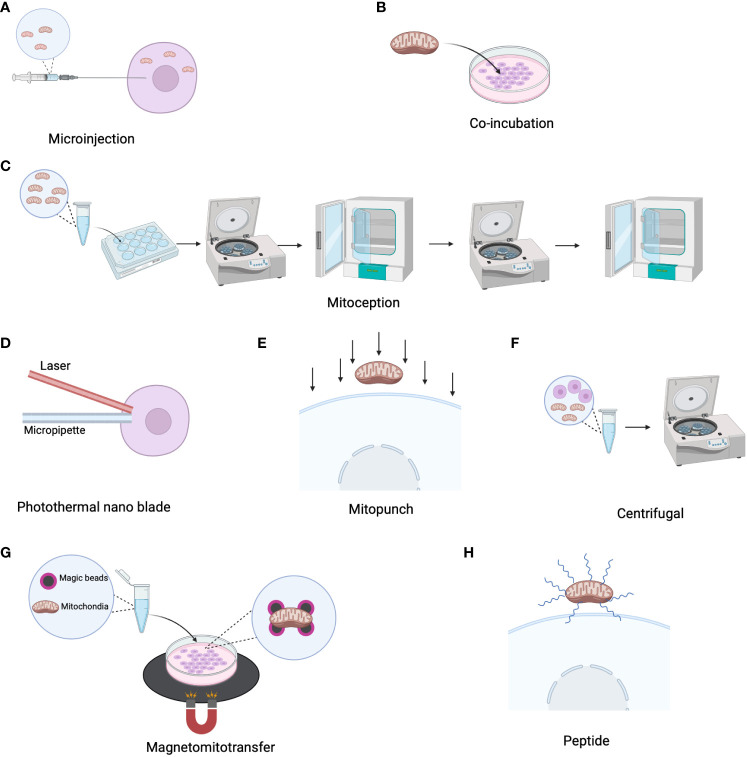
Mitochondrial transmission strategy. **(A)** Transfer isolated mitochondria to recipient cells via microinjection. **(B)** Co-incubate isolated mitochondria with recipient cells to facilitate mitochondria transfer. **(C)** Mitoception: Add mitochondrial suspension to the entire culture surface, followed by centrifugation of the culture plate at 1500 g for 15 minutes at 4°C. Subsequently, place them in a 37°C cell culture incubator and perform a second centrifugation under the same conditions after two hours. Incubate in the incubator for 24 hours. **(D)** Photo-nanotube: Employ laser pulses to induce pressure-driven liquid flow in the micropipette, facilitating the delivery of mitochondria into the recipient cytoplasm. **(E)** Mitopunch: Utilize a pressure-driven mitochondrial transfer device. **(F)** Centrifugal: Prepare mitochondria by centrifuging at 1500 g for 5 minutes without requiring additional incubation. **(G)** Magnetomitotransfer: Perform mitochondrial transfer of magnetically labeled mitochondria with the aid of magnetic plates. **(H)** Enhance mitochondrial transfer through peptide encapsulation on the mitochondrial surface.

Mitochondria can not only naturally transfer from cell to cell, but they can also be extracted, concentrated, and modified before transplantation into various cells for the treatment of metabolic diseases associated with mitochondrial dysfunction. In 1988, King et al. applied microinjection (**Figure 2A**) to inject isolated mitochondria containing chloramphenicol-resistant markers encoded by mitochondrial DNA (mtDNA) into human 143BTK- and HT1080-6TG cells, and found evidence of endogenous mitochondrial DNA replacement (69). However, microinjection can damage cells and is less effective than co-culture (70). In 2014, Tomoya Kitani discovered that genetically labeled mitochondria can be transferred to homogeneic and xenogeneic cells through simple co-culture (**Figure 2B**) (61). However, the internalization of isolated mitochondria into cells is challenging due to their relatively large size and negatively charged surface. To address this issue, Andres Caicedo developed a technique called MitoCeption, which involves slowly adding a suspension of mitochondria to recipient cells in a culture dish, followed by centrifugation and incubation in a culture incubator to enhance the efficiency of mitochondrial transplantation (**Figure 2C**) (71). However, this method has limitations as direct fusion and transfer between transferred and endogenous mitochondria are not possible, and the transfer efficiency is generally low. To overcome these challenges, Ting-Hsiang Wu and colleagues invented a novel method for transferring isolated mitochondria from donor mammalian cells to recipient cells using a photothermal nanoblade (**Figure 2D**), which consists of a 532 nm nanosecond pulsed laser, a nanoblade delivery micropipette, and a microscope. Under laser pulsation, pressure drives liquid flow in the micropipette, delivering mitochondria into the recipient cytoplasm (72). As this method requires complex laser and optical systems to operate, the team developed “MitoPunch” based on the technology of photothermal nanoblades, a pressure-driven mitochondrial transfer device that can simultaneously deliver isolated mitochondria to many target mammalian cells. This is a force-based mitochondrial transfer method previously described, which can generate stable isolated mitochondrial recipient (SIMR) cells that retain exogenous mtDNA permanently (**Figure 2E**). The MitoPunch device is versatile and has low assembly costs, making it a higher-throughput and more user-friendly system (73). While these methods offer high purity, the transplantation efficiency is relatively low. Mi Jin Kim developed a simple centrifugation-based method for mitochondrial transfer that can be applied to any type of cell (**Figure 2F**) (74). This method successfully transfers isolated mitochondria to target cells (74). Magnetic bead labeled mitochondria with the assistance of magnetic plates have also been found to be a method to improve the efficiency of mitochondrial transfer (**Figure 2G**) (75). Other substances have been found to facilitate the uptake of mitochondria by cells, such as cell-penetrating peptides Pep-1 (**Figure 2H**), biocompatible polymers (dextran‐triphenylphosphonium), which promote the uptake of mitochondria by recipient cells (76, 77). SS31, a novel cell-permeable antioxidant peptide, shows the ability to effectively mitigate and decrease the production of reactive oxygen species within isolated mitochondria (78). Furthermore, research finding indicates that doxycycline, an antibiotic drug, exhibit the potential to augment mitochondrial transfer within cancer cells, thereby facilitating the restoration of mitochondrial function (79).

## 5 Therapeutic potential of mitochondrial transfer in metabolic diseases

**Figure 3:**
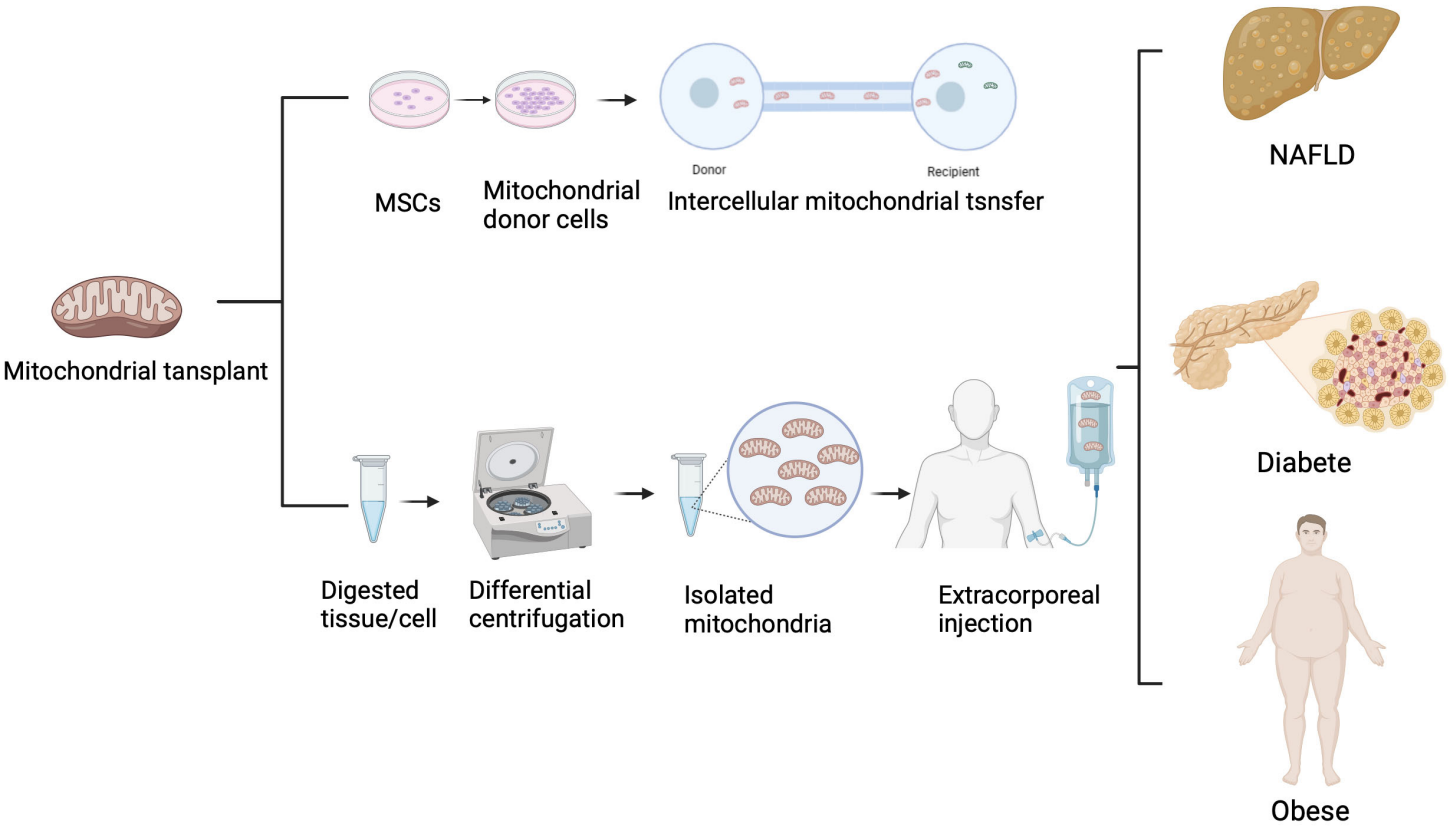
Treatment of metabolic diseases with mitochondrial transfer.

Cell therapy, as an emerging therapeutic approach, can be applied to the treatment of various diseases, including cancer, immune system disorders, and genetic diseases. However, the immunological rejection of xenogeneic cells severely limits their application. Many metabolic diseases are closely associated with mitochondrial dysfunction or mutations in mitochondrial DNA. Moreover, compared to cell therapy, mitochondria have lower immunogenicity, making them a potential tool for the treatment of various diseases through mitochondrial transfer. Clinical trials have demonstrated the application of mitochondrial transplantation in cardiovascular treatment. Pediatric patients, susceptible to myocardial ischemia-reperfusion injury following surgery, benefit from the direct injection of autologous mitochondria into the ischemic myocardium. These transplanted mitochondria enhance recovery and cell viability for up to 28 days without triggering adverse immune reactions (80). Retrospective studies involving pediatric patients with Ischemia-Reperfusion Injury (IRI) showcase the effectiveness of mitochondrial transplantation in reducing recovery duration and mitigating cardiovascular incidents (81). Furthermore, mitochondrial transplantation shows promise in addressing infertility and contributing to advancements in reproductive medicine. Despite the absence of conducted clinical trials for mitochondrial transplantation in metabolic diseases, numerous *in vivo* and *in vitro* experiments highlight its potential in treating such disorders (**Figure 3**).

### 5.1 Applications of mitochondrial transfer in the treatment of diabetes and its complications

Complications of diabetes, including cardiovascular disease, neuropathy, nephropathy, and retinopathy, have been extensively documented (82–85). The utilization of mitochondria for the treatment of diabetes has also been widely explored. Rackham et al. found that under co-culture conditions, adipose-derived mesenchymal stem cells can transfer mitochondria to human pancreatic beta cells, thereby enhancing their insulin secretion function (86).

Mitochondrial dysfunction caused by T2DM increases the susceptibility of the myocardium to ischemia-reperfusion injury. Doulamis et al. studied the therapeutic effect of mitochondria isolated from diabetic or non-diabetic rats on ischemia-reperfusion injury in the hearts of diabetic rats. The results showed that mitochondria derived from diabetic rats produced significantly less ATP than those from non-diabetic rats. Furthermore, the study found that mitochondrial transplantation significantly enhanced post-ischemic myocardial function recovery and significantly reduced cardiomyocyte damage in diabetic hearts (87). A similar conclusion was drawn from another study where offspring rats exposed to pre-gestational diabetes and a high-fat diet displayed cardiac dysfunction, mitochondrial dysfunction, and compromised cellular bioenergetics. The extracellular transplantation of healthy mitochondria into cardiac cells of these offspring rats significantly improved cellular respiration in male rats and reduced apoptosis, but increased apoptosis in female rats (88).

Diabetic nephropathy (DN) is a common complication of T2DM that reduces the quality of life of patients. Yuan et al. found that mitochondrial transfer from mesenchymal stem cells (MSCs) to macrophages (Mφ) can inhibit inflammation in the kidneys of diabetic nephropathy mice by activating PGC-1. Under co-culture conditions of MSCs and Mφ, mitochondria from MSCs (MSCs-Mito) were transferred to Mφ, improving mitochondrial function in Mφ and alleviating kidney damage in DN mice (89).

Diabetes-associated cognitive impairment (DACI) poses a risk to patient health and increases the risk of major cardiovascular events, cardiovascular death, and all-cause mortality (90). Ma et al. evaluated the cognitive behavior of db/db mice using the Morris water maze test to investigate whether transplantation of platelet-derived mitochondria (Mito-Plt) could improve DACI. The results showed that Mito-Plt injected into the lateral ventricle were internalized into hippocampal neurons. One month after Mito-Plt transplantation, DACI in db/db mice was alleviated, mitochondrial quantity increased, mitochondrial function recovered, oxidative stress and neuronal apoptosis were reduced, and the accumulation of Aβ and Tau in the hippocampus was decreased. In conclusion, Mito-Plt transplantation alleviated cognitive impairment and mitochondrial dysfunction in db/db mice. This method may have potential applications in the treatment of DACI (91).

The above evidence suggests that mitochondrial transfer has therapeutic effects in diabetes and its complications including diabetic nephropathy, diabetic cardiomyopathy, and diabetes-associated cognitive impairment.

### 5.2 Application of mitochondrial transfer in the treatment of NAFLD

In 2017, Fu et al. reported the utilization of isolated mitochondria from liver cancer cells as a therapeutic agent for treating high-fat diet-induced fatty liver in mice. After intravenous injection of mitochondria into mice, serum transaminase activity and cholesterol levels decreased in a dose-dependent manner. Furthermore, this mitochondrial treatment reduced lipid accumulation and oxidative damage in the livers of fatty liver mice, improving energy production in liver cells and restoring liver function. This treatment strategy provides a potential new approach for the treatment of NAFLD (92). Similarly, Paliwal et al. used mitochondria isolated from normal rat chest muscles to treat metabolic syndrome rats induced by a high-fat diet (HFD) and streptozotocin (STZ). The results showed that mitochondrial transplantation reduced the levels of systolic and diastolic blood pressure, decreased blood glucose levels, and significantly decreased blood lipid levels in metabolic syndrome rats. Histopathological analysis showed improvements in alanine aminotransferase (ALT) and aspartate aminotransferase (AST) levels, and significant restoration of liver morphology. In addition, enhanced mitochondrial biogenesis, reduced oxidative stress and inflammatory markers were observed (93). Hsu et al. demonstrated that bone marrow-derived mesenchymal stem cells (MSCs) can also treat non-alcoholic steatohepatitis (NASH). They transplanted bone marrow-derived MSCs into the livers of a non-alcoholic steatohepatitis mouse model, resulting in improvements in liver lipid content, tissue inflammation, and fibrosis. Co-culture of liver cells and MSCs revealed that mitochondria were transferred from MSCs to liver cells through TNTs. Therefore, MSCs can contribute to lipid breakdown in liver cells by providing oxidative capacity through the donation of mitochondria to liver cells, promoting the recovery of metabolic and tissue damage induced by NASH (94). Furthermore, Fu and colleagues injected isolated mitochondria from human hepatocellular carcinoma (Hep G2 cells) into the livers of fatty liver mice and found that this treatment improved liver energy production, reduced liver lipid accumulation and oxidative damage, and restored liver cell activity (92). Therefore, mitochondrial transfer may be a promising approach for the treatment of NAFLD.

### 5.3 Applications of mitochondrial transfer in the treatment of obesity

To date, there have been no reports on the use of mitochondrial transfer for the treatment of obesity, but studies have shown a relationship between obesity and mitochondrial transfer. Clemente-Postigo et al. found that pro-inflammatory cytokines such as interferon (IFN)-γ and lipopolysaccharide (LPS) activate macrophages toward an M1 polarization in white adipose tissue (WAT) of obese patients, while anti-inflammatory cytokines such as interleukin (IL)-4 and IL-13 drive macrophages toward an M2 polarization (95). Brestoff et al. also found that macrophages polarized to an M1 phenotype when treated with IFN-γ and LPS, resulting in reduced expression of the heparan sulfate (HS) biosynthetic genes in BV2 cells and impaired mitochondrial transfer from adipocytes to macrophages. Furthermore, in obese patients induced by a high-fat diet (HFD), both M1 and M2 macrophage populations showed reduced mitochondrial transfer (42). These studies suggest that reduced mitochondrial transfer from adipocytes to macrophages is a feature of obesity, and therefore, mitochondrial transfer may be a potential approach for the treatment of obesity.

## 6 Challenges

Although mitochondrial transplantation holds great potential, there are several challenges that hinder its clinical application. First, mitochondria possess their own DNA, which can be inherited through cytoplasmic transmission, raising safety concerns. During preclinical assessments of mitochondrial donation, a significant safety concern is the gradual increase in initial low levels of “mtDNA carryover” that co-transmits with the nuclear genome during mitochondrial transplantation, potentially increasing during pregnancy and posing a risk of severe mtDNA disease in offspring (96–98).

Second, xenogeneic mitochondrial transplantation raises concerns about immunological reactions, although the reaction is relatively low compared to cell transplantation. Xenogeneic mitochondria can activate innate immunity, with pattern recognition receptors (such as Toll-like receptors and Nod-like receptors) recognizing damage-associated molecular patterns (DAMPs) (99, 100). Transplantation of xenogeneic mitochondria into various tissues has been associated with increased markers of self-immunity and inflammation (101). Lin et al. further elucidated that mitochondrial stimulation directly triggers inflammatory responses in endothelial cells, leading to adhesion and activation of alloreactive T cells, ultimately increasing the risk of alloreactive transplantation rejection (102). Autologous and transplanted mitochondria may also interact with each other.

Next, it is crucial to take into account the potential impact of the isolation procedure on the structure and functionality of mitochondria obtained from cells or tissues. Research has demonstrated that this process elevates mitochondrial stress levels and promotes the generation of free radicals (103). Additionally, the isolation procedure may lead to the disruption of the outer mitochondrial membrane, facilitating the entry of cytochrome C into the buffer solution. Consequently, this disruption hampers oxygen consumption and ATP production (104). Hence, it is imperative to conduct various assays, including the utilization of fluorescent probes and electron microscopy, to thoroughly evaluate mitochondrial integrity and function prior to engaging in mitochondria transplantation (105–107).

Moreover, the efficiency of mitochondrial transplantation is often suboptimal. Despite the emergence of various methods to improve transfer efficiency, mitochondrial function is still affected. The commonly used differential centrifugation-based method suffers from low purity and is time-consuming (60, 108). However, recent advantages have introduced techniques like MitoPunch, which can simultaneously transfer mitochondria to 100,000 or more recipient cells, significantly improving the throughput and efficiency compared to existing methods (71). Continued advancements in techniques will increase the efficiency of future mitochondrial transplantation and make this approach more viable.

Additionally, the storage of mitochondria remains a challenge (109). Isolated mitochondria can maintain activity for 1-2 hours on ice. However, even with the use of preservatives, the integrity of the mitochondrial outer membrane is damaged when stored at -80°C (110). While storing mitochondria with cryoprotectants like DMSO and trehalose preserves outer membrane integrity, it impairs mitochondrial function (111, 112). Therefore, it is crucial to develop storage methods that maintain the stability and functionality of mitochondria (108).

Another consideration is whether transplanted mitochondria can effectively engage with other cellular organelles to ensure their functionality. Notably, previous studies have provided evidence that upon transplantation, exogenous mitochondria undergo fusion with endogenous counterparts (113). This transplantation process facilitates the integration of transferred mitochondria into the mitochondrial network of the recipient cell. Consequently, there exists the potential for transplanted mitochondria to establish functional associations with other organelles within the recipient cells (114).

Addressing these challenges will pave the way for the realization of mitochondrial transplantation as a viable therapeutic approach for treating various diseases including metabolic diseases.

## 7 Conclusion

This article provides a comprehensive review of the relationship between metabolic diseases and mitochondria, as well as the role of mitochondrial transfer in their treatment. However, despite the well-established association between mitochondrial dysfunction and metabolic diseases, there is a scarcity of studies exploring the therapeutic potential of mitochondrial transplantation or transfer. Most studies focus primarily on common diseases such as diabetes and NAFLD, leaving other metabolic diseases with limited research. In addition, most of the studies on mitochondrial transfer have been conducted in animal models, and their translation into clinical applications is yet to be realized.

Mitochondria exhibit low immunogenicity, offer convenient accessibility, and present minimal ethical concerns. As a result, employing mitochondria for the treatment of metabolic diseases holds remarkable advantages. Despite the limited number of clinical trials on mitochondrial therapy (115), advancements in technology hold immense potential for its extensive application, particularly in the treatment of metabolic disorders.

## Author contributions

RC: Writing – original draft. JC: Writing – original draft, Conceptualization, Funding acquisition, Supervision, Writing – review & editing.
